# Intention to initiate antiretroviral therapy (ART) among people living with HIV in China under the scaling-up of ART: the role of healthcare workers’ recommendations

**DOI:** 10.1186/s12913-019-4143-9

**Published:** 2019-05-16

**Authors:** Qiangsheng He, Xuan Du, Huifang Xu, Lirui Fan, Remina Maimaitijiang, Yanan Wu, Chun Hao, Jinghua Li, Yuantao Hao, Jing Gu

**Affiliations:** 1 Department of Medical Statistics, School of Public Health, Sun Yat-sen University, No.74 Zhongshan Road 2, Guangzhou, 510080 Guangdong People’s Republic of China; 2 Department of HIV Prevention, Guangzhou Centre for Disease Control and Prevention, Guangzhou, 510440 People’s Republic of China; 3 Sun Yat-sen Global Health Institute, Institute of State Governance, Sun Yat-sen University, Guangzhou, 510275 People’s Republic of China

**Keywords:** HIV/AIDS, Antiretroviral therapy, Intention, Healthcare workers, China

## Abstract

**Background:**

The early initiation of antiretroviral therapy (ART) for people living with HIV (PLWH) benefits both individuals and societies. However, little is known about the intention to initiate ART among PLWH in China in the context of a scaling-up of treatment or how the recommendations of healthcare workers affect this intention.

**Methods:**

A total of 451 ART-naïve PLWH were recruited from communities in Guangzhou, China for this study. Data were collected by trained physicians via face-to-face interviews. Logistic regression models were fitted for the data analyses.

**Results:**

Of the participants, 93.8% were male, 72.7% were infected via homosexual behaviour and 68.5% reported an intention to initiate ART. In the latter category, 77.8, 41.9 and 20.0% of respondents received strong recommendations to initiate ART from healthcare workers at the Centres for Disease Control and Prevention (CDC), community healthcare centres and non-governmental organisations (NGOs), respectively. After adjusting for potential confounders, depression, anxiety and strong recommendations from healthcare workers at the CDC and NGOs correlated significantly with ART intention. In the adjusted final hierarchical logistic regression model, the duration of infection [multivariate odds ratio (OR_m_) = 0.30, *p* < 0.001], route of HIV infection (OR_m_ = 0.18, *p* < 0.01), infection status of the current spouse/regular sex partner (OR_m_ = 0.21–0.23, *p* < 0.01), anxiety (OR_m_ = 2.44–2.65, *p* < 0.05) and strong recommendations from CDC physicians (OR_m_ = 3.67, *p* < 0.01) or NGOs workers (OR_m_ = 3.67, *p* < 0.01) were independently associated with the ART intention, whereas a recommendation from a community healthcare centre physician was not.

**Conclusions:**

In Guangzhou, the prevalence of ART intention was below the 90–90-90 targets. Further studies aimed at an in-depth understanding and encouragement of health care workers’ perceptions regarding early ART are warranted as a means of scaling up new ART strategies.

## Background

Human immunodeficiency virus (HIV) remains a major public health threat. By the end of 2017, 758,610 people in China were reportedly living with HIV and 134,512 new HIV infections had occurred during that year [1]. Early antiretroviral therapy (ART) can effectively suppress the replication of the HIV virus, delay the progression of disease, reduce HIV/AIDS-related mortality and decrease the risk of HIV transmission [2–7]. In 2015, increasing evidence led the World Health Organisation (WHO) to recommend the initiation of ART in all people living with HIV (PLWH), irrespective of their CD4 cell counts [8]. Implementation of the new guideline will provide significant contribution to the achievement of the UNAIDS “90–90-90” target: 90% of all PLWH will know their HIV status, 90% of all people with HIV diagnosed will receive sustained ART and 90% of all people receiving ART will have viral suppression by 2020 [9]. Achievement of these 90–90-90 targets has the potential to end the AIDS epidemic by 2030, and accelerating ART initiation is crucial to achieve the second 90% target.

In China, the National Free Antiretroviral Treatment Program (NFATP) was started in 2003 [10]. Following updated WHO ART guidelines, China revised the ART policy and further expanded free ART services [11, 12]. From 2008, PLWH with CD4 cell counts below 350 became eligible for treatment; since 2014, the treatment threshold was CD4 cell counts below 500 [13]; and recently since 2016, China updated its ART guideline according to the new WHO strategy and has provided free ART for all PLWH [14].

Despite the benefits of ART, various countries have reported unsatisfactory levels of coverage [9]. The ART coverage rates worldwide and in China were 53 and 67%, respectively, in 2015 [9, 15]. Previous research identified various factors associated with ART initiation, including those related to socio-demographic status (e.g., age, gender, marital status) [16–18] and HIV status (e.g., duration of HIV infection, route of HIV infection, CD4 count) [18–21]. Although the literature also suggests that psycho-social factors such as depression, anxiety and social support also affected ART initiation, no consensus has been reached. For example, Tao et al. reported positive associations of depression and anxiety with earlier ART initiation among PLWH in China [22], whereas studies conducted elsewhere reported that mental health problems, stigma and a lack of social support led to delays in ART initiation [23]. Furthermore, most recent research was conducted before new ART strategies were implemented. Therefore, a few unaddressed issues remain. For example, it remains uncertain whether PLWH with a wider range of CD4 cell counts would want to initiate treatment in the context of a scaling up of ART. Furthermore, the effects of various factors on the treatment intentions of PLWH are unknown. These questions have not been well studied in China or other countries, despite their critical importance to the practical implementation of new ART guidelines.

Research has shown that physicians’ recommendations may serve as a crucial stimulus for ART initiation [24–26]. One European study of men who have sex with men reported that a lack of recommendation from a physician was cited as the most common reason for a lack of ART initiation [27]. Furthermore, a Chinese study identified a physician’s recommendation as the main reason underlying the decision to initiate ART among PLWH with high CD4 cell counts [25]. Nonetheless, the above findings were based on qualitative studies, and the influences of healthcare workers’ recommendations on ART initiation among PLWHs have not been investigated quantitatively.

In China, PLWH with confirmed HIV infection are followed by district-level Centres for Disease Control and Prevention (CDC) or Community Healthcare Centres (CHCs; sub-district level) according to the population size of local HIV/AIDS, according to the National Practical Guideline for the Follow-up and Management of PLWH. Furthermore, ART-related information is delivered to PLWH by healthcare workers at CDC or CHCs [28]. In practice, non-governmental organisations (NGOs) which provide services to HIV-related populations may also be involved in the process of communication with PLWH [29]. As new ART strategies have only been implemented recently, the firmness of ART-related recommendations provided to PLWH may vary across healthcare workers from different organisations. However, no previous study has quantitatively compared the effects of recommendations from healthcare workers affiliated with different organisations on intentions toward ART in China.

This study aimed to explore the intentions regarding ART initiation among PLWH in Guangzhou, China and to identify the effects of various factors, especially healthcare workers’ recommendations, on these intentions after the implementation of the new ART guideline. As intention and behaviour are strongly correlated [30, 31], the findings of this study could contribute to a better understanding of the ART initiation behaviours of PLWH and provide evidence to support a better implementation of the scaling-up of ART in China.

## Methods

### Study design and participants

This cross-sectional study was conducted in Guangzhou, the capital city of Guangdong Province. By the end of 2015, 7311 HIV cases had been reported in Guangzhou (unpublished statistical data, 2015) and 5056 PLWH (69.2%) were receiving ART (Guangzhou Yearbook, 2016). Six out of 11 districts in Guangzhou with relatively high HIV prevalence rates were purposively selected for this study. The study population comprised ART-naïve PLWH who were aged ≥18 years, clinically eligible for ART and could provide informed consent. Participants who did not finish the interview were excluded. All potential participants were recruited from the local CDC and CHCs after attending CD4 count testing sessions between June 2016 and April 2017. Participants who provided informed consent then participated in face-to-face interviews via structured questionnaires, which were administered by trained physicians in private settings. Identifiable information such as name and ID was not recorded to ensure the anonymity of interview. A total of 466 eligible participants were recruited, of whom 451 (96.8%) completed the interview. According to the recommendation on sample size of multivariate analysis models, the minimum sample size should be 10 to 20 times the number of independent variables included in the model [32, 33]. Our sample size met the need of analysis (no more than 20 independent variables in one model). Ethical approval for the study was obtained from the School of Public Health, Sun Yat-sen University.

### Measurements

Background information for the study included socio-demographic (gender, age, ethnicity, education level, current marital status, health insurance status, origin) and HIV-related characteristics (i.e., duration of HIV infection, route of HIV infection, infection status of current spouse/regular sex partner, most recent CD4 counts, history of AIDS-related clinical symptoms).

Characteristics of the psycho-social status, including depression, anxiety, stigma and social support, were also measured. Probable depression was assessed using the Patient Health Questionnaire (PHQ-9) [34–37], and scores of 5, 10, 15 and 20 were used as cut-off points for mild, moderate, moderately severe and severe depression, respectively [34]. In this study, the Cronbach’s alpha value of the PHQ-9 was 0.909, and participants were grouped into 3 depressive symptom categories: normal (score 0–4), mild (5–9) and moderate-severe (≥10) [37]. The Generalised Anxiety Disorder 7 (GAD-7) scale was used to measure current anxiety [38, 39]. In this study, the Cronbach’s alpha value for the GAD-7 was 0.946. We categorised participants as exhibiting normal (score 0–4), mild (5–9), and moderate-severe (≥10) anxiety symptoms [38]. Self-perceived stigma was assessed using the 9-item Chinese Courtesy Stigma Scales (CCSSs) [40], which yield scores ranging from 5 to 45. A higher score indicates a higher level of self-perceived stigma (Cronbach’s alpha = 0.90 in this study). Finally, the 12-item Perceived Social Support Scale (PSSS) has been used in previous studies targeting PLWH [41]. This scale yields possible scores ranging from 12 to 84, with higher scores indicating a higher level of perceived social support. In this study, the Cronbach’s alpha of the PSSS was 0.919.

Recommendations to initiate ART from healthcare workers at CHCs, the CDC and NGOs were documented. We asked the participants whether each type of healthcare worker had recommended the initiation of ART. The response options included ‘no recommendation’, ‘little recommendation’, ‘some recommendation’, ‘strong recommendation’ and ‘not applicable (i.e., have not previously had contact with this type of healthcare worker)’. For the data analysis, these responses were combined into 3 groups: ‘weak recommendation’ (no/little recommendation), ‘strong recommendation’ (some/strong recommendation) and ‘not applicable’.

One statement (‘When will you initiate ART?’) was used to assess the dependent variable (the intention to initiate ART). The response options included ‘right now’, ‘only when my CD4 counts drop to a certain level’, ‘not ready for ART now’, ‘have never thought about it (ART)’ and ‘other’. A response of ‘right now’ was considered to indicate the intention to initiate ART.

### Data analysis

Univariate logistic regression models were fitted to assess the associations between the studied background variables and ART intention. Variables with a *p* value < 0.1 were included as candidates in the multivariate stepwise logistic regression models [42] (Table 2). Both psycho-social status variables and recommendations from different types of healthcare workers were set as key independent variables, and multivariate analyses adjusted for background variables identified as significant in the multivariate analysis were performed (Table 3). Hierarchical logistic regression models were fitted to examine the independent effects of psycho-social status variables and recommendations from healthcare workers on ART intentions (Table 4). Initially, background variables were selected (stepwise) using Model 1. Next, Model 2 was constructed by the stepwise selection of a block of psycho-social variables for addition to Model 1 (which was forced to stay in the model). Finally, Model 3 was constructed similarly using the variable of recommendations from healthcare workers. All analyses were performed using SPSS for Windows, version 22.0 (SPSS, Chicago, IL, USA). A *p* value < 0.05 was considered statistically significant.

## Results

### Background characteristics

Table 1 lists the profiles of all participants. Most participants were male (93.8%) and of Han ethnicity (93.6%). The average age was 32.91 years [standard deviation (SD) = 10.40 years], and 64.3% of participants were younger than 35 years. Nearly 40.0% of participants had attained a university-level or higher education and 59.9% were single. Most (64.5%) had health insurance, and 26.1% were local residents of Guangzhou. More than half (52.5%) of the participants were newly diagnosed as HIV-positive within 1 month, 72.7% were infected via homosexual behaviour and 11.5% reported that their current spouse or regular sex partners were HIV-positive. Furthermore, 53.9% of participants had a most recent CD4 cell count of < 350 and 18.6% had AIDS-related clinical symptoms.

**Table 1:** Profiles of all participants (*n* = 451)

	Number	Percent
Socio-demographic characteristics		
Male	423	93.8
Age (mean: 32.91, sd: 10.40)		
18–	105	23.3
25–	185	41.0
35–80	161	35.7
Han ethnicity	422	93.6
Education level		
Junior high or below	128	28.4
Senior high school	142	31.5
College or above	181	40.1
Current marital status		
Single	270	59.9
Married or cohabitation	123	27.3
Others (Married separation/Divorce/Others)	58	12.9
Having health insurance	291	64.5
Origin		
Local (Guangzhou)	117	26.1
Other places of Guangdong Province	107	23.8
Other Provinces	225	50.1
HIV-related characteristics		
Duration of HIV infection (months since HIV diagnosis)		
<1	237	52.5
1–12	78	17.3
>12	136	30.2
Route of HIV infection		
Heterosexual behaviour	104	23.1
Homosexual behaviour	328	72.7
Injecting drug use/uncertain	19	4.2
Infection status of current spouse/regular sex partners		
Negative	57	12.6
Positive	52	11.5
Don’t know	150	33.3
Without partner	192	42.6
Most recent CD4 counts		
<=350	243	53.9
>350	208	46.1
Ever had AIDS-related clinical symptoms		
No	367	81.4
Yes	84	18.6
Psycho-social status		
Depression (mean: 7.76, sd: 5.84)		
Normal (0–4)	153	33.9
Mild (5–9)	159	35.3
Moderate-severe (10–27)	139	30.8
Psycho-social status		
Anxiety (5.63, 4.99)		
Normal (0–4)	213	47.2
Mild (5–9)	158	35.0
Moderate-severe (10–21)	80	17.7
Perceived stigma (mean: 31.56, sd: 6.81)		
Social support (mean: 57.18, sd: 14.16)		
Healthcare workers’ recommendations to initiate ART		
From physicians at CHCs		
Weak recommendation	94	20.8
Strong recommendation	189	41.9
Not applicable	168	37.3
From physicians at the CDC		
Weak recommendation	67	14.9
Strong recommendation	351	77.8
Not applicable	33	7.3
From healthcare workers at NGOs		
Weak recommendation	101	22.4
Strong recommendation	90	20.0
Not applicable	260	57.6

Not applicable: participants have not previously had contact with this type of healthcare worker

CHCs: Community Healthcare Centres

NGOs: non-governmental organizations

CDC: the Centres for Disease Control and Prevention

### Psycho-social status and healthcare workers’ recommendations to initiate ART

The analysis of psycho-social factors revealed that 30.8% of participants had moderate-severe depression, while 17.7% had moderate-severe anxiety (Table 1). The mean perceived stigma and social support scores were 31.56 (SD = 6.81) and 57.18 (SD = 14.16), respectively. Overall, 398 participants (88.2%) had received a strong recommendation to initiate ART from a healthcare worker of any type, and 41.9, 77.8 and 20.0% had received strong recommendations to initiate ART from physicians at CHCs, physicians at the CDC and healthcare workers at NGOs, respectively.

### Intention to initiate ART and associated background factors

Overall, 309 participants (68.5%) reported an intention to initiate ART ‘right now’. In a multivariate analysis (Table 2), participants with a longer duration of HIV infection [multivariate OR (OR_m_) = 0.19–0.49, *p* < 0.05), those infected via a non-sexual route (e.g., injected drug use; OR_m_ = 0.16, *p* < 0.01), those with an HIV-positive spouse/regular sex partner or who lacked a regular partner (OR_m_ = 0.31 and 0.32, respectively, *p* < 0.05) and those with a higher recent CD4 count (OR_m_ = 0.58, *p* < 0.05) were less likely than others to express an intention to initiate ART.

**Table 2:** Associations between background factors and ART intention

	%	Univariate		Multivariate	
		OR_u_	95% CI	OR_m_	95% CI
Socio-demographic characteristics					
Male	69.3	1.69	(0.78,3.67)	N.S.	
Age				N.S.	
18–	70.5	1.00			
25–	66.5	0.83	(0.50,1.40)		
35–80	69.6	0.96	(0.56,1.64)		
Han ethnicity	69.0	1.36	(0.62,2.96)	N.S.	
Education level				N.S.	
Junior high or below	68.0	1.00			
Senior high school	62.7	0.79	(0.48,1.31)		
College or above	73.5	1.31	(0.80,2.15)		
Current marital status					
Single	65.6	1.00		N.S.	
Married or cohabitation	74.0	1.49	(0.93,2.40)+		
Others	70.7	1.27	(0.68,2.35)		
Having health insurance	71.1	1.41	(0.93,2.13)	N.S.	
Origin				N.S.	
Local (Guangzhou)	66.7	1.00			
Other places of Guangdong Province	68.2	1.07	(0.61,1.88)		
Other Provinces	69.8	1.15	(0.72,1.86)		
HIV-related variables					
Duration of HIV infection (months since HIV diagnosis)					
<1	83.5	1.00		1.00	
1–12	66.7	**0.39**	**(0.22,0.71)** ^**^	**0.49**	**(0.27,0.91)** ^*^
>12	43.4	**0.15**	**(0.09,0.25)** ^***^	**0.19**	**(0.11,0.31)** ^***^
Route of HIV infection					
Heterosexual behaviour	70.2	1.00		1.00	
Homosexual behaviour	69.8	0.98	(0.61,1.59)	0.92	(0.53,1.59)
Injecting drug use/uncertain	36.8	**0.25**	**(0.09,0.69)** ^**^	**0.16**	**(0.05,0.52)** ^**^
Infection status of current spouse/regular sex partners					
Negative	78.9	1.00		1.00	
Positive	63.5	0.46	(0.20,1.09)^+^	**0.31**	**(0.12,0.80)** ^*^
Don’t know	76.7	0.88	(0.42,1.84)	0.62	(0.27,1.41)
Without partner	60.4	**0.41**	**(0.20,0.82)** ^*^	**0.32**	**(0.14,0.70)** ^**^
Most recent CD4 counts					
<=350	77.0	1.00		1.00	
>350	58.7	**0.43**	**(0.28,0.64)** ^***^	**0.58**	**(0.37,0.93)** ^*^
Ever had AIDS-related clinical symptoms				N.S.	
No	67.3	1.00			
Yes	73.8	1.37	(0.80,2.33)		

*OR*_*u*_: odds ratio of univariate logistic regression models

*OR*_*m*_: odds ratio of multivariate logistic regression models using all variables with *p* < 0.1 in the univariate analysis as candidates (stepwise)

^+^: *p* < 0.1; ^*^:*p* < 0.05; ^**^: *p* < 0.01; ^***^: *p* < 0.001

ORs with *p* < 0.05 are in bold

N.S.: Non-significant in the multivariate analysis

### Associations between psycho-social factors, healthcare workers’ recommendations and ART intention

After adjusting for significant background variables, participants with moderate-severe depression (adjusted OR [OR_a_] = 2.85, *p* < 0.001) or mild or moderate-severe anxiety (OR_a_ = 2.50 and 2.29, respectively, *p* < 0.05) were more likely to express an intention to initiate ART (Table 3). Regarding recommendation-related variables, participants who received strong recommendations from healthcare workers at the CDC (OR_a_ = 4.77, *p* < 0.001) or NGOs (OR_a_ = 4.89, *p* < 0.001) were more likely to initiate ART, compared to those who received weak recommendations.

**Table 3:** Associations between psycho-social factors, healthcare workers’ recommendations and ART intention

		Univariate		Multivariate	
	%	OR_u_	95% CI	OR_a_	95% CI
Psycho-social status					
Depression					
Normal (0–4)	57.5	1.00		1.00	
Mild (5–9)	67.9	1.56	(0.99,2.48)^+^	1.58	(0.94,2.66)^+^
Moderate-severe (10–27)	81.3	**3.21**	**(1.88,5.47)** ^***^	**2.85**	**(1.57,5.16)** ^***^
Anxiety					
Normal (0–4)	57.3	1.00		1.00	
Mild (5–9)	78.5	**2.72**	**(1.71,4.34)** ^***^	**2.50**	**(1.49,4.18)** ^***^
Moderate-severe (10–21)	78.8	**2.76**	**(1.52,5.04)** ^***^	**2.29**	**(1.17,4.45)** ^*^
Perceived stigma		1.00	(0.97,1.03)	1.00	(0.97,1.03)
Social support		1.01	(0.99,1.03)	1.02	(1.00,1.03)^+^
Healthcare workers’ recommendations to initiate ART					
From physicians at CHCs					
Weak recommendation	58.5	1.00		1.00	
Strong recommendation	58.2	0.99	(0.6,1.63)	1.21	(0.69,2.11)
Not applicable	85.7	**4.26**	**(2.35,7.72)** ^***^	**2.94**	**(1.53,5.66)** ^***^
From physicians at the CDC					
Weak recommendation	40.3	1.00		1.00	
Strong recommendation	76.1	**4.71**	**(2.73,8.13)** ^***^	**4.77**	**(2.51,9.05)** ^***^
Not applicable	45.5	1.24	(0.53,2.86)	1.26	(0.48,3.33)
From healthcare workers at NGOs					
Weak recommendation	46.5	1.00		1.00	
Strong recommendation	80	**4.60**	**(2.40,8.78)** ^***^	**4.89**	**(2.37,10.08)** ^***^
Not applicable	73.1	**3.12**	**(1.94,5.03)** ^***^	**2.78**	**(1.60,4.84)** ^***^

*OR*_*u*_: odds ratio of univariate logistic regression models;

*OR*_*a*_:odds ratios of adjusted logistic regression models’ adjusting for background variables which were multivariately significant in Table 2

^+^: *p* < 0.1; ^*^:*p* < 0.05; ^**^: *p* < 0.01; ^***^: *p* < 0.001

ORs with *p* < 0.05 are in bold

### Hierarchical logistic regression analyses

The results of hierarchical logistic regression analyses are reported in Table 4. In the final model, participants with a longer duration of infection (OR_m_ = 0.30, *p* < 0.001), those infected through a non-sexual route (OR_m_ = 0.18, *p* < 0.01) and those with an HIV-positive spouse/regular sex partner (OR_m_ = 0.21, *p* < 0.01) were less likely than others to express an intention to initiate ART. The reverse was true for participants with mild or moderate-severe anxiety (OR_m_ = 2.65 and 2.44, respectively, *p* < 0.05). In the final analysis, strong recommendations from CDC physicians (OR_m_ = 3.67, *p* < 0.01) and NGO workers (OR_m_ = 3.67, *p* < 0.01) were independently associated with an intention to initiate ART.

**Table 4:** Odds ratios of hierarchical models of ART intention

	Model 1	Model 2	Model 3
Background			
Duration of HIV infection (months since HIV diagnosis)			
<1	1.00	1.00	1.00
1–12	**0.49 (0.27,0.91)** ^*^	0.54 (0.29,1.02)^+^	0.79 (0.40,1.57)
>12	**0.19 (0.11,0.31)** ^***^	**0.21 (0.12,0.35)** ^***^	**0.30 (0.16,0.55)** ^***^
Route of HIV infection			
Heterosexual behaviour	1.00		
Homosexual behaviour	0.92 (0.53,1.59)	0.95 (0.54,1.66)	0.79 (0.43,1.46)
Injecting drug use/uncertain	**0.16 (0.05,0.52)** ^**^	**0.16 (0.05,0.54)** ^**^	**0.18 (0.05,0.62)** ^**^
Infection status of current spouse/regular sex partners			
Negative	1.00	1.00	1.00
Positive	**0.31 (0.12,0.80)** ^*^	**0.32 (0.12,0.85)** ^*^	**0.21 (0.07,0.60)** ^**^
Don’t know	0.62 (0.27,1.41)	0.65 (0.28,1.49)	0.60 (0.25,1.42)
Without partner	**0.32 (0.14,0.70)** ^**^	**0.31 (0.14,0.70)** ^**^	**0.23 (0.10,0.54)** ^**^
Most recent CD4 counts			
<=350	1.00	1.00	1.00
>350	**0.58 (0.37,0.93)** ^*^	**0.60 (0.37,0.96)** ^*^	0.62 (0.37,1.03)^+^
Psycho-social status			
Anxiety	N.A.		
Normal (0–4)		1.00	1.00
Mild (5–9)		**2.50 (1.49,4.18)** ^***^	**2.65 (1.52,4.62)** ^***^
Moderate-severe (10–21)		**2.29 (1.17,4.45)** ^*^	**2.44 (1.19,4.98)** ^*^
Healthcare workers’ recommendations to initiate ART			
From physicians at CHCs	N.A.	N.A.	
Weak recommendation			1.00
Strong recommendation			0.53 (0.26,1.11)^+^
Not applicable			1.19 (0.53,2.71)
From physicians at the CDC	N.A.	N.A.	
Weak recommendation			
Strong recommendation			**3.67 (1.62,8.33)** ^**^
Not applicable			1.03 (0.31,3.44)
From healthcare workers at NGOs	N.A.	N.A.	
Weak recommendation			
Strong recommendation			**3.67 (1.58,8.52)** ^**^
Not applicable			2.00 (1.00,4.02)^+^
**-2LL(d.f.)**	473.043(8)^***^	458.218(10)	395.715(17)
**∆-2LL(d.f.) comparison with Model 1**	N.A.	−14.825^***^ (2)	N.A.
**∆-2LL(d.f.) comparison with Model 2**	N.A.	N.A.	−47.406^***^ (6)

^+^: *p* < 0.1; ^*^:*p* < 0.05; ^**^: *p* < 0.01; ^***^: *p* < 0.001

NA: Not applicable

-2*LL*(d.f.): −2 log likelihood (degree of freedom)

ORs with *p* < 0.05 are in bold

## Discussion

In this study, the observed prevalence of ART intention, 68.5%, was similar to the rates reported in China (62.7–65.2%) [25, 43] and other countries, such as South Africa (78.4%), Thailand (78.8%) and Kenya (60.2%) [44–46]. Although the implementation of new ART strategies has been translated into increased treatment coverage, a gap remains between the current treatment intentions and 90–90-90 targets [47]. Our study identified a positive association between strong recommendations from CDC physicians and NGO workers and the intention to initiate ART. These findings may inform future interventions intended to promote ART intention and uptake behaviours among PLWH.

Healthcare workers play a crucial role in HIV treatment promotion and are considered a main source of ART-related information for PLWH [24, 25, 48]. In this study, however, we found that recommendations from CDC physicians, but not from CHC physicians, facilitated ART intentions. Furthermore, our results showed a lower likelihood of ART intention among participants who were followed by CHC physicians. This relationship was demonstrated by hierarchical models which demonstrated that the recommendations from CHC physicians were non-significant when CDC physicians were included as a variable.

Both CHCs and CDC physicians conduct follow-ups of PLWH. Possibly, the latter would have a stronger influence than the former on the ART decisions of PLWH. In practice, the responsibilities of CHCs physicians in community health-related services are more comprehensive (e.g., health education, both communicable and noncommunicable disease prevention), and these professionals are relatively less well-trained in the provision of HIV-related services, compared to CDC physicians who usually play a more focused and sometimes exclusive role in HIV/AIDS prevention and control [49–51]. The literature supports the view that for CHCs physicians, a heavy workload and lack of training could lead to unfamiliarity with the new guidelines, improper perceptions of the benefits of early ART and too little time to communicate sufficiently with PLWH [24, 51–53]. However, CHCs workers are directly responsible for the follow-up and management of ART-naïve PLWH in some areas of China. This warrants the training of CHCs physicians and innovations in HIV case management programs in community settings.

We additionally found that recommendations from NGOs workers were positively associated with ART initiation. The unique characteristics of NGOs have enabled a significant and supportive role in the expansion of HIV services [29]. Specifically, NGOs act as a bridge between healthcare workers and PLWH and are thus able to spread correct knowledge to PLWH, influence perceptions about early ART initiation and thereby accelerate treatment uptake. In this study, more than half (57.6%) of the participants had not had previous contact with NGOs, suggesting a potentially greater role for these organisations in ART promotion in China.

In our study, PLWH with current depression/anxiety were more willing to initiate ART, and anxiety was independently associated with ART intention. These findings differed from our expectations and are inconsistent with previous studies reporting that mental health problems could delay ART initiation [54, 55]. However, our findings are consistent with recent studies that demonstrated increased ART uptake among PLWH with depression or anxiety [22, 56]. Likely, these depressed and/or anxious PLWH were more concerned about their health status and were more likely to seek health services [22]. Additionally, studies have reported that early ART initiation could help to ease the mental health symptoms of PLWH [56, 57]. These results suggest that early ART could be promoted successfully among PLWH with symptoms of depression/anxiety [56].

Our study results also indicated negative associations of the duration of HIV infection and relationships with HIV-infected sex partners with the ART intention. Our finding was consistent with previous reports that people with a longer duration of HIV infection tended to be healthier, with a higher CD4 cell count and characteristics that did not meet earlier ART criteria. Such patients may not perceive a need for treatment and worry about side effects, drug resistance and lifestyle inconvenience and would thus defer the initiation of ART until their disease has progressed to a more serious condition [24, 25, 53]. PLWH whose sex partners were also HIV-positive might also be less motivated to initiate ART, compared to those who wish to protect their HIV-negative partners [58]. Future health programs intended to promote ART should focus on these PLWH.

This study had some limitations. First, we only investigated PLWH in one city, which restricts the generalisation of our findings across all PLWH in China. Second, the study outcome was behavioural intention rather than actual ART initiation behaviour. Despite a close relationship, intention and behaviour remain separate, and the consistency of this relationship may be influenced by additional factors. Third, this was a cross-sectional study. Therefore, the results could not establish a causal relationship between the independent variables and the outcome. Finally, as data were self-reported, there would be recall bias; and because of social desirability, the prevalence of ART intention might be over-estimated.

## Conclusions

The Joint United Nations Programme on HIV and AIDS has set an ambitious goal of ending the AIDS epidemic by 2030 through the 90–90-90 strategy [47]. Our findings indicate that a significant number of PLWH were unwilling to initiate ART immediately under the scaling-up strategies. In the future, training and coaching programmes for healthcare workers that focus on PLWH with certain characteristics (e.g., a longer duration of infection) are greatly warranted.

## Abbreviations

AIDS: Acquired immune deficiency syndrome
ART: Antiretroviral therapy
CDC: Centres for Disease Control and Prevention
CHCs: Community healthcare centres
HIV: Human immunodeficiency virus
N.S.: Non-significant in the multivariate analysis
NA: Not applicable
NGOs: Non-governmental organisations
OR_a_: adjusted odds ratio
OR_m_: multivariate odds ratio
PLWH: People living with HIV
SD: standard deviation
WHO: the World Health Organisation

## References

[1] NCAIDS, NCSTD, and China CDC (2018). Update on the AIDS/STD epidemic in December 2017. Chin J AIDS STD.

[2] Lundgren D, Babiker AG, Gordin F, Emery S, Sharma S, Avihingsanon AC, Cooper DA, Tkenheuer GF, Llibre JM, Moli-Na JM (2015). Initiation of antiretroviral therapy in early asymptomatic HIV infection. N Engl J Med.

[3] Grinsztejn B, Hosseinipour MC, Ribaudo HJ, Swindells S, Eron J, Chen YQ, Wang L, Ou SS, Anderson M, McCauley M (2014). Effects of early versus delayed initiation of antiretroviral treatment on clinical outcomes of HIV-1 infection: results from the phase 3 HPTN 052 randomised controlled trial. Lancet Infect Dis.

[4] Samji H, Cescon A, Hogg RS, Modur SP, Althoff KN, Buchacz K, Burchell AN, Cohen M, Gebo KA, Gill MJ (2013). Closing the gap: increases in life expectancy among treated HIV-positive individuals in the United States and Canada. PLoS One.

[5] Le T, Wright EJ, Smith DM, He W, Catano G, Okulicz JF, Young JA, Clark RA, Richman DD, Little SJ (2013). Enhanced CD4+ T-cell recovery with earlier HIV-1 antiretroviral therapy. N Engl J Med.

[6] Danel C, Moh R, Gabillard D, Badje A, Le Carrou J, Ouassa T, Ouattara E, Anzian A, Ntakpe J-B, Minga A (2015). A trial of early Antiretrovirals and isoniazid preventive therapy in Africa. N Engl J Med.

[7] Cohen MS, Chen YQ, McCauley M, Gamble T, Hosseinipour MC, Kumarasamy N, Hakim JG, Kumwenda J, Grinsztejn B, Pilotto JHS (2011). Prevention of HIV-1 infection with early antiretroviral therapy. N Engl J Med.

[8] World Health Organization: Guideline on when to start antiretroviral therapy and on pre-exposure prophylaxis for HIV: World Health Organization; 2015. 26598776

[9] UNAIDS: UNAIDS data 2017. In*.*: Joint United Nations Programme on HIV/AIDS (UNAIDS) Geneva, Switzerland; 2017.

[10] Zhang F, Haberer JE, Wang Y, Zhao Y, Ma Y, Zhao D, Yu L, Goosby EP (2007). The Chinese free antiretroviral treatment program: challenges and responses. Aids.

[11] Tang Z, Pan SW, Ruan Y, Liu X, Su J, Zhu Q, Shen Z, Zhang H, Chen Y, Lan G (2017). Effects of high CD4 cell counts on death and attrition among HIV patients receiving antiretroviral treatment: an observational cohort study. Sci Rep.

[12] D-c Z, Yi W, Ye M, Yan Z, Zhang Y, Wu Y-S, Xia L, Elizabeth A, Liu Z-F, Zhang F-J (2012). Expansion of China’s free antiretroviral treatment program. Chin Med J.

[13] Zun-You WU: HIV/AIDS in China: Beyond the Number**s**: Springer; 1st ed; 2017.6.

[14] Ministry of Health and National Population and Family Planning Commission of the People’s Republic of China:Ministry of Health and National Population and Family Planning Commission of the People’s Republic of China’s announcement on standard of antiretroviral treatment (in Chinese). [http://www.gov.cn/xinwen/2016-06/15/content_5082505.htm].

[15] Zun-You WU. The progress and challenges of promoting HIV/AIDS 90-90-90 strategies in China. Chinese Journal of Disease Control & Prevention. 2016.

[16] Gari S, Martin-Hilber A, Malungo JR, Musheke M, Merten S (2014). Sex differentials in the uptake of antiretroviral treatment in Zambia. AIDS Care.

[17] Mujugira A, Celum C, Thomas KK, Farquhar C, Mugo N, Katabira E, Bukusi EA, Tumwesigye E, Baeten JM (2014). Delay of antiretroviral therapy initiation is common in east African HIV-infected individuals in serodiscordant partnerships. J Acquir Immune Defic Syndr.

[18] Liu Y, Ruan Y, Vermund SH, Osborn CY, Wu P, Jia Y, Shao Y, Qian HZ (2015). Predictors of antiretroviral therapy initiation: a cross-sectional study among Chinese HIV-infected men who have sex with men. BMC Infect Dis.

[19] Boyer S, Iwuji C, Gosset A, Protopopescu C, Okesola N, Plazy M, Spire B, Orne-Gliemann J, McGrath N, Pillay D (2016). Factors associated with antiretroviral treatment initiation amongst HIV-positive individuals linked to care within a universal test and treat programme: early findings of the ANRS 12249 TasP trial in rural South Africa. AIDS Care.

[20] Kiertiburanakul S, Boettiger D, Lee MP, Omar SF, Tanuma J, Ng OT, Durier N, Phanuphak P, Ditangco R, Chaiwarith R (2014). Trends of CD4 cell count levels at the initiation of antiretroviral therapy over time and factors associated with late initiation of antiretroviral therapy among Asian HIV-positive patients. J Int AIDS Soc.

[21] Mao L, de Wit JB, Kippax SC, Prestage G, Holt M (2015). Younger age, recent HIV diagnosis, no welfare support and no annual sexually transmissible infection screening are associated with nonuse of antiretroviral therapy among HIV-positive gay men in Australia. HIV Med.

[22] Tao J, Vermund SH, Lu H, Ruan Y, Shepherd BE, Kipp AM, Amico KR, Zhang X, Shao Y, Qian HZ (2017). Impact of depression and anxiety on initiation of antiretroviral therapy among men who have sex with men with newly diagnosed HIV infections in China. AIDS Patient Care STDs.

[23] Kahn TR, Desmond M, Rao D, Marx GE, Guthrie BL, Bosire R, Choi RY, Kiarie JN, Farquhar C (2013). Delayed initiation of antiretroviral therapy among HIV-discordant couples in Kenya. AIDS Care.

[24] Bolsewicz K, Debattista J, Vallely A, Whittaker A, Fitzgerald L (2015). Factors associated with antiretroviral treatment uptake and adherence: a review. Perspectives from Australia, Canada, and the United Kingdom. AIDS Care.

[25] Zhang Q, Tang Z, Sun H, Cheng P, Qin Q, Fan Y, Huang F (2015). Acceptability of early anti-retroviral therapy among HIV-infected people in Anhui province in China. AIDS Care.

[26] Bukenya D, Wringe A, Moshabela M, Skovdal M, Ssekubugu R, Paparini S, Renju J, McLean E, Bonnington O, Wamoyi J (2017). Where are we now? A multicountry qualitative study to explore access to pre-antiretroviral care services: a precursor to antiretroviral therapy initiation. Sexually transmitted infections.

[27] Marcus U, Hickson F, Weatherburn P, Furegato M, Breveglieri M, Berg RC, Schmidt AJ, Network F (2015). Antiretroviral therapy and reasons for not taking it among men having sex with men (MSM)¡ªResults from the European MSM internet survey (EMIS). PLoS One.

[28] China CDC:Practice Guidelines for Follow-up and management of people living with HIV/AIDS in China. 2016.

[29] Semitala FC, Camlin CS, Wallenta J, Kampiire L, Katuramu R, Amanyire G, Namusobya J, Chang W, Kahn JG, Charlebois ED, et al. Understanding uptake of an intervention to accelerate antiretroviral therapy initiation in Uganda via qualitative inquiry. J Int AIDS Soc. 2017;20. 10.1002/jia2.25033 PMC5810312 29206357

[30] Webb TL, Sheeran P (2006). Does changing behavioral intentions engender behavior change? A meta-analysis of the experimental evidence. Psychol Bull.

[31] Alavi M, Micallef M, Fortier E, Dunlop AJ, Balcomb AC, Day CA, Treloar C, Bath N, Haber PS, Dore GJ (2015). Effect of treatment willingness on specialist assessment and treatment uptake for hepatitis C virus infection among people who use drugs: the ETHOS study. J Viral Hepat.

[32] Vittinghoff E, McCulloch CE (2007). Relaxing the rule of ten events per variable in logistic and cox regression. Am J Epidemiol.

[33] Ogundimu EO, Altman DG, Collins GS (2016). Adequate sample size for developing prediction models is not simply related to events per variable. J Clin Epidemiol.

[34] Kroenke K, Spitzer RL, Williams JB (2001). The PHQ-9: validity of a brief depression severity measure. J Gen Intern Med.

[35] Chen S, Chiu H, Xu B, Ma Y, Jin T, Wu M, Conwell Y (2010). Reliability and validity of the PHQ-9 for screening late-life depression in Chinese primary care. INTERNATIONAL JOURNAL OF GERIATRIC PSYCHIATRY.

[36] Wang W, Bian Q, Zhao Y, Li X, Wang W, Du J, Zhang G, Zhou Q, Zhao M (2014). Reliability and validity of the Chinese version of the patient health questionnaire (PHQ-9) in the general population. Gen Hosp Psychiatry.

[37] Sanchez SE, Puente GC, Atencio G, Qiu C, Yanez D, Gelaye B, Williams MA (2013). Risk of spontaneous preterm birth in relation to maternal depressive, anxiety, and stress symptoms. J Reprod Med.

[38] Spitzer RL, Kroenke K, Williams JBW, Lowe B (2006). A brief measure for assessing generalized anxiety disorder - the GAD-7. Arch Intern Med.

[39] Tong X, An D, McGonigal A, Park S-P, Zhou D (2016). Validation of the generalized anxiety Disorder-7 (GAD-7) among Chinese people with epilepsy. Epilepsy Res.

[40] Liu H, Xu Y, Sun Y, Dumenci L (2014). Measuring HIV stigma at the family level: psychometric assessment of the Chinese courtesy stigma scales (CCSSs). PLoS One.

[41] Su X, Lau JT, Mak WW, Chen L, Choi K, Song J, Zhang Y, Zhao G, Feng T, Chen X (2013). Perceived discrimination, social support, and perceived stress among people living with HIV/AIDS in China. AIDS Care.

[42] Cleophas TJ, Zwinderman AH, Cleophas TF (2002). Statistics Applied to Clinical Trials.

[43] Jiang H-H, Lü F, He H-J, Zhang D-D, Zeng G, Xu P, Ma F-C, Xin Q-Q, Cheng J, Pan X-H (2013). Acceptability status of early antiretroviral therapy among HIV-positive men who have sex with men. Zhonghua yu fang yi xue za zhi [Chinese journal of preventive medicine].

[44] Garrett N, Norman E, Leask K, Naicker N, Asari V, Majola N, Karim QA, Karim SSA. Acceptability of early antiretroviral therapy among south African women. AIDS Behav. 2017:1–7. 10.1007/s10461-017-1729-2 PMC5565727 28224322

[45] Maek-a-nantawat W, Phanuphak N, Teeratakulpisarn N, Pakam C, Kanteeranon T, Chaiya O, Mansawat T, Teeratakulpisarn S, Nonenoy S, Ananworanich J (2014). Attitudes toward, and interest in, the test-and-treat strategy for HIV prevention among Thai men who have sex with men. AIDS Care.

[46] Heffron R, Ngure K, Mugo N, Celum C, Kurth A, Curran K, Baeten JM: Willingness of Kenyan HIV-1 serodiscordant couples to use antiretroviral based HIV-1 prevention strategies. *Journal of acquired immune deficiency syndromes (1999)* 2012, 61(1):116. 10.1097/QAI.0b013e31825da73f PMC3427394 22595872

[47] The Joint United Nations Programme on HIV/AIDS (UNAIDS) (2014). 90–90-90: an ambitious treatment target to help end the AIDS epidemic.

[48] Christopoulos KA, Olender S, Lopez AM, Lekas H-M, Jaiswal J, Mellman W, Geng E, Koester KA (2015). Retained in HIV care but not on antiretroviral treatment: a qualitative patient-provider dyadic study. PLoS Med.

[49] DENG B, LIU Y, W-H LAI (2011). Human resource at community level for HIV/AIDS Preventionand control in Sichuan Province. Journal of Preventive Medicine Information.

[50] Ma F, Xu P, Jiang H-H, Zhang D, Meng S, Zeng G, He H-J. Research on supportability of workers related to HIV /AIDS prevention practice in community health service centers. Medicine & Society. 2013.

[51] Wang Y, Peng XU, Zhou Y. Problems and solutions for HIV/AIDS prevention and control in China. Chinese Journal of Public Health Management. 2016.

[52] Krastinova E, Seng R, Yeni P, Viard J-P, Vittecoq D, Lascoux-Combe C, Fourn E, Pahlavan G, Delfraissy JF, Meyer L (2013). Is clinical practice concordant with the changes in guidelines for antiretroviral therapy initiation during primary and chronic HIV-1 infection? The ANRS PRIMO and COPANA cohorts. PLoS One.

[53] Fehr J, Nicca D, Goffard J-C, Haerry D, Schlag M, Papastamopoulos V, Hoepelman A, Skoutelis A, Diazaraque R, Ledergerber B (2016). Reasons for not starting antiretroviral therapy in HIV-1-infected individuals: a changing landscape. Infection.

[54] Murray LK, Semrau K, McCurley E, Thea DM, Scott N, Mwiya M, Kankasa C, Bass J, Bolton P (2009). Barriers to acceptance and adherence of antiretroviral therapy in urban Zambian women: a qualitative study. AIDS Care.

[55] De R, Bhandari S, Roy S, Bhowmik A, Rewari B, Guha S (2013). Factors responsible for delayed enrollment for anti-retroviral treatment. J Nepal Health Res Counc.

[56] Velloza J, Celum C, Haberer JE, Ngure K, Irungu E, Mugo N, Baeten JM, Heffron R (2017). Depression and ART initiation among HIV serodiscordant couples in Kenya and Uganda. AIDS Behav.

[57] Chan BT, Weiser SD, Boum Y, Haberer JE, Kembabazi A, Hunt PW, Martin JN, Mocello AR, Bangsberg DR, Tsai AC (2015). Declining prevalence of probable depression among patients presenting for antiretroviral therapy in rural Uganda: the role of early treatment initiation. AIDS Behav.

[58] Patel RC, Odoyo J, Anand K, Stanford-Moore G, Wakhungu I, Bukusi EA, Baeten JM, Brown JM (2016). Facilitators and barriers of antiretroviral therapy initiation among HIV discordant couples in Kenya: qualitative insights from a pre-exposure prophylaxis implementation study. PLoS One.

